# Targeting CD38 in Subclinical Antibody-mediated Rejection in HLA-incompatible Kidney Transplantation: A Case Report

**DOI:** 10.1097/TXD.0000000000001685

**Published:** 2024-07-18

**Authors:** Ondrej Viklicky, Petra Hruba, Marek Novotny, Martin Kment, Matej Roder, Philip F. Halloran, Georg A. Böhmig

**Affiliations:** 1 Department of Nephrology, Institute for Clinical and Experimental Medicine, Prague, Czech Republic.; 2 Transplant Laboratory, Institute for Clinical and Experimental Medicine, Prague, Czech Republic.; 3 Department of Pathology, Institute for Clinical and Experimental Medicine, Prague, Czech Republic.; 4 Department of Immunogenetics, Institute for Clinical and Experimental Medicine, Prague, Czech Republic.; 5 Alberta Transplant Applied Genomics Centre, Edmonton, AB, Canada.; 6 Division of Nephrology and Dialysis, Department of Medicine III, Medical University Vienna, Vienna, Austria.

Presensitized patients with donor-specific antibodies (DSAs) are at increased risk of antibody-mediated rejection (AMR) of kidney allografts.^1^ The long-term consequences of AMR are serious because currently available therapeutic options lack lasting effectiveness.^2^ Targeting plasma cells to counter antibody production may hold promise for treating AMR.^3^ Daratumumab is a fully humanized monoclonal antibody directed against CD38, a glycoprotein expressed at high levels on plasma cells and, in addition, natural killer cells, which are suggested to act as effector cells in AMR.^4,5^ Several case reports suggested its efficacy in the treatment of AMR in patients with and without multiple myeloma.^6-8^ Timing of such therapy is of significant concern because serum creatinine alone cannot distinguish subclinical injury, and innovative tools are necessary for more precise diagnostics. Here, we describe a case of severe DSA rebound associated with subclinical AMR by histology and molecular assessments effectively reversed by a 6-mo course of daratumumab.

## CASE DESCRIPTION

The patient is a 55-y-old man who has undergone fourth kidney transplantation in November 2022. The deceased brain death donor was a 61-y-old man with well-preserved kidney function (estimated glomerular filtration rate [eGFR]: 2.4 mL/s). Despite a high level of sensitization (calculated panel-reactive antibody 91%), there was only a single anti-HLA class II DSA present (specificity: DQA1*01:02/DQB1*06:02; mean fluorescence intensity [MFI]: 4200). Both actual cytotoxic and flow T and B crossmatch were negative. A detailed description of the HLA typing of all donors and recipient is given in **Table S1 (SDC,** http://links.lww.com/TXD/A680).

**FIGURE 1.**
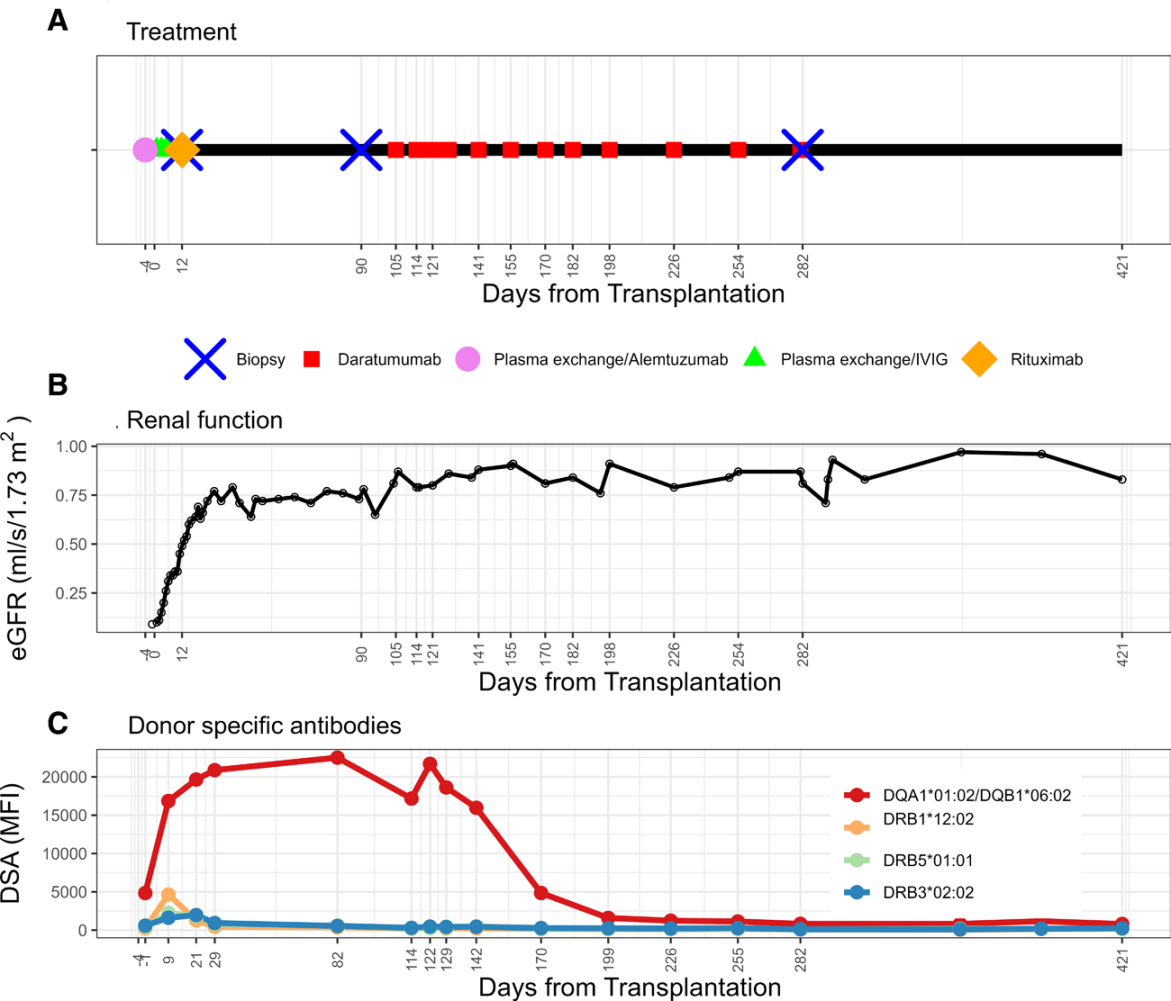
Clinical course of the case. A, Therapy during the follow-up. B, Renal function. C, DSAs. DSA, donor-specific antibody; eGFR, estimated glomerular filtration rate; MFI, mean fluorescence intensity.

A single plasma exchange immediately before surgery was performed for desensitization. A single intravenous dose of alemtuzumab (30 mg) was applied perioperatively as induction (Figure 1A). Peritransplant desensitization included 5 plasma exchanges every other day, along with a high dose of IVIG (2 g/kg in total).^9^ For maintenance immunosuppression, tacrolimus (target trough level 8–12 µg/L), mycophenolate mofetil (2000 mg), and tapered prednisone were administered. Infection prophylaxis consisted of valganciclovir and trimethoprim/sulfamethoxazole. Graft function developed immediately (Figure 1B).

At postoperative day (POD) 9, Luminex-based single antigen testing revealed a substantial increase in anti-HLA reactivity. Specifically, a significant rebound of the preformed donor-specific anti-DQA1*01:02/DQB1*06:02 (16 000 MFI) was noticed along with the presence of 3 different DSA-targeting DR antigens (DRB1*12:02, DRB5*01:01 and DRB3*02:02, maximum MFI 4600; Figure 1C). Tacrolimus trough levels exceeded the target range (18.4 µg/L).

**FIGURE 2.**
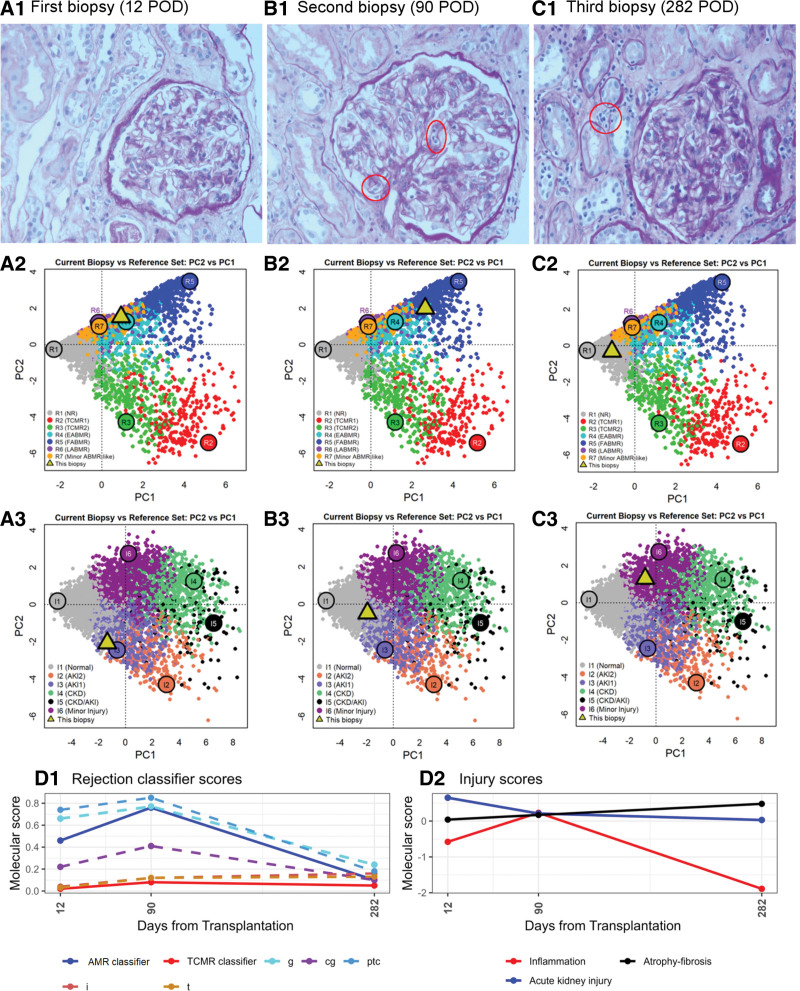
Histologic (A1, B1, C1) and MMDx results (A2, A3, B2, B3, C2, C3, D1, and D2) of index and surveillance biopsies. Histopathologic examination revealed acute tubular necrosis in the first biopsy, active AMR in the second biopsy (glomerulitis shown by red circles), and resolution of g and remaining mild ptc only (shown by a red circle) in the third biopsy. Biopsies assignments to specific rejection and injury archetypal phenotypes, as defined by MMDx-based analysis, are depicted (yellow triangles) in PC plots (PC1 vs PC2) based on a reference set of 5087 biopsies.^10^ Colored circles indicate the locations of 6 rejection archetype centers: R1: no rejection; R2: TCMR1; R3: TCMR2; R4: early-stage AMR; R5: fully developed AMR; R6: late-stage AMR; R7, minor AMR and 6 injury archetype centers—R1: normal; R2: AKI1; R3: AKI2; R4: CKD; R5: CKD/AKI; and R6: Minor CKD. The development of molecular scores between index and follow-up biopsies. D1, Rejection classifier scores. D2, Injury scores. Dashed lines, molecular scores for particular Banff histologic lesions. AKI, acute kidney injury; AMR, antibody-mediated rejection; cg, transplant glomerulopathy; CKD, chronic kidney disease; g, glomerulitis; i, interstitial inflammation; MMDx, Molecular Microscope Diagnostic System; PC, principal component; ptc, peritubular capillaritis; t, tubulitis; TCMR, T cell–mediated rejection.

On POD 12, acute tubular necrosis without microvascular inflammation (MVI) or capillary C4d staining was present in the graft biopsy (Figure 2A1). However, Molecular Microscope Diagnostic System (MMDx) assessment showed a finding of moderate early stage AMR (Figure 2A2 and A3). Serum creatinine at the time of biopsy was 210 µmol/L and tacrolimus trough levels 12 µg/L. Donor-derived cell-free DNA (dd-cfDNA; Prospera) testing revealed a profound level of graft injury, with fractions increasing to 3.21% (negative result defined as <1%). Clearly, early after transplantation, the dd-cfDNA assessment cannot rule out other peritransplant injuries. However, considering the presence of molecular AMR and DSA rebound, we planned the administration of rituximab (1 g) on top of the induction protocol. However, its administration was complicated by a serious allergic reaction, and patient thus received a steroid bolus. The patient was discharged home with a serum creatinine of 165 µmol/L (eGFR of 0.66 mL/s). Another dd-cfDNA assessment at POD 30 revealed an ongoing allograft injury (7.97%) despite stable kidney graft function.

At POD 90, a surveillance biopsy revealed considerable MVI (glomerulitis [g2] and peritubular capillaritis [ptc2]), suggesting morphologic AMR activity (Figure 2B1). MMDx showed severe fully developed AMR (Figure 2B2 and B3). The immunodominant DSAs against DQA1*01:02/DQB1*06:02 remained consistently elevated (15 000 MFI). Tacrolimus level was 10.5 µg/L, and virology screening revealed BK virus replication (23 000 copies/ mL); however, the biopsy did not show features of polyomavirus nephropathy and graft function remained stable. As a consequence, mycophenolate mofetil was transiently reduced to 500 mg/d.

Based on the ongoing presence of class II DSAs, worsening of histological and molecular features of AMR, and positive dd-cfDNA, we initiated rescue treatment with daratumumab at POD 115 when the BK virus plasma load decreased. Daratumumab was given subcutaneously (1800 mg/dose) 11 times during a period of 6 mo. Initially, the daratumumab dose was given every week 4 times, followed by every other week 5 times and finally monthly 2 times (Figure 1A).

A second surveillance biopsy was performed at POD 282, coinciding with the last daratumumab dose. The extent of MVI (g0, ptc1) was below the diagnostic criteria for definite AMR (Figure 2C1). MMDx analysis indicated complete resolution of rejection-related scores (Figure 2C2 and C3). The current graft function is stable (eGFR of 0.8 mL/s), and the dd-cfDNA test performed 1 y after transplantation yielded a negative result (0.08%). There were no rehospitalizations for infection complications, but 1 month after completion of treatment, the patient did experience a mild COVID-19 disease treated with remdesivir.

During daratumumab treatment, DSA levels declined, eventually reaching positivity thresholds, and remained negative until the last measurement at POD 421 (Figure 1C). The changes in DSA levels were accompanied by a substantial decrease in levels of non-DSA HLA antibodies. Detailed descriptions of molecular assessments of kidney allograft on days 12, 90 and 285 are given in Figure 2A–D.

## DISCUSSION

Despite the high risk of AMR, HLA-incompatible transplantation may remain the only chance for transplantation for some patients if acceptable mismatch programs are not available or a long waiting time for a compatible graft is not realistic. Our case suggests the efficacy of targeting CD38 to counter active AMR and points out the critical role innovative diagnostic methods have in HLA-incompatible transplantation. The kidney transplantation was performed across low levels of preformed anti-HLA DQ reactivity associated with the negative flow and cytotoxic crossmatches before surgery. Despite desensitization, we detected molecular features of AMR, which were associated with DSA rebound early posttransplantation. In AMR, molecular assessment may be a more precise diagnostic option than conventional histology,^11,12^ particularly in HLA-incompatible kidney transplantation when patients receive profound induction immunosuppression, which mitigates but does not definitely alter the alloimmune response. MMDx has been implemented in US transplant centers and is currently available in our center for routine assessments.

Daratumumab was given as a salvage therapy at 3 mo when DSA levels persisted unchanged, surveillance biopsy confirmed AMR by both histology and MMDx, and dd-cfDNA indicated profound injury. The prognosis of such a graft was considered to be poor despite a mostly subclinical presentation of rejection. A total daratumumab dose was given over a limited course of 6 mo, primarily to mitigate infection risks and limit treatment costs. This approach was successful because it led to DSA elimination achieved after only 6 doses, and, notably, a second surveillance biopsy after 6 mo showed normalization of histology and molecular assessments. Current follow-up has been uneventful, with no evidence of DSA rebound, stable kidney graft function and negative dd-cfDNA.

In conclusion, a 6-mo course of daratumumab was well tolerated and demonstrated efficacy in converting DSA rebound and normalizing histology, molecular microscope, and dd-cfDNA assessments. Future systematic interventional trials are needed to establish the efficacy and safety of this approach in larger cohorts.

## Supplementary Material


