# Supplementary material for: Targeting CD38 in Subclinical Antibody-mediated Rejection in HLA-incompatible Kidney Transplantation: A Case Report
Source: Transplant Direct. 2024 Jul 18;10(8):e1685. doi: 10.1097/TXD.0000000000001685 (PMC11259386; doi:10.1097/TXD.0000000000001685)
Supplement: Supplementary file 1 [file txd-10-e1685-s001.pdf]

Table S1. Detailed description of HLA typing of all donors and recipient.

HLA locus	A	B	C	DRB1	DRB3,4,5	DQB1	DQA1	DPB1	DPA1
Patient	2	60; 61	2	4; 11	DRB3				
1. donor	2; 32	49; 60		13	DRB3				
2. donor	26; 29	38; 44		07; 11	DRB3; DRB4				
3. donor	2; 24	61; 44		04; 11	DRB3; DRB4	2; 7		4	
4. donor	02:01	07:02; 46:01	01:02; 07:02	12:02; 15:01	DRB3*02:02; DRB5*01:01	03:01; 06:02	01:02; 06:01	02:01; 04:01	01:03
